# Implications of reconstruction protocol for histo-biological characterisation of breast cancers using FDG-PET radiomics

**DOI:** 10.1186/s13550-018-0466-5

**Published:** 2018-12-29

**Authors:** Nicolas Aide, Thibault Salomon, Cécile Blanc-Fournier, Jean-Michel Grellard, Christelle Levy, Charline Lasnon

**Affiliations:** 1 Nuclear Medicine Department, University Hospital, Caen, France; 2 INSERM 1199 ANTICIPE, Normandy University, Caen, France; 3 Pathology Department, François Baclesse Cancer Centre, Caen, France; 4 Biostatistics and Clinical Research Unit, François Baclesse Cancer Centre, Caen, France; 5 Breast Cancer Unit, François Baclesse Cancer Centre, Caen, France; 6 Nuclear Medicine Department, François Baclesse Cancer Centre, 3 Avenue du Général Harris, BP 45026 Cedex 5, 14076 Caen, France

**Keywords:** ^18^F-FDG-PET, Breast cancer, Heterogeneity, Radiomics, Reconstruction

## Abstract

**Background:**

The aim of this study is to determine if the choice of the ^18^F-FDG-PET protocol, especially matrix size and reconstruction algorithm, is of importance to discriminate between immunohistochemical subtypes (luminal versus non-luminal) in breast cancer with textural features (TFs).

**Procedures:**

Forty-seven patients referred for breast cancer staging in the framework of a prospective study were reviewed as part of an ancillary study. In addition to standard PET imaging (PSF_WholeBody_), a high-resolution breast acquisition was performed and reconstructed with OSEM and PSF (OSEM_breast_/PSF_breast_). PET standard metrics and TFs were extracted. For each reconstruction protocol, a prediction model for tumour classification was built using a random forests method. Spearman coefficients were used to seek correlation between PET metrics.

**Results:**

PSF_WholeBody_ showed lower numbers of voxels within VOIs than OSEM_breast_ and PSF_breast_ with median (interquartile range) equal to 130 (43–271), 316 (167–1042), 367 (107–1221), respectively (*p* < 0.0001). Therefore, using LifeX software, 28 (59%), 46 (98%) and 42 (89%) patients were exploitable with PSF_WholeBody_, OSEM_breast_ and PSF_breast_, respectively.

On matched comparisons, PSF_breast_ reconstruction presented better abilities than PSF_wholeBody_ and OSEM_breast_ for the classification of luminal versus non-luminal breast tumours with an accuracy reaching 85.7% as compared to 67.8% for PSF_wholeBody_ and 73.8% for OSEM_breast_. PSF_breast_ accuracy, sensitivity, specificity, PPV and NPV were equal to 85.7%, 94.3%, 42.9%, 89.2%, 60.0%, respectively. Coarseness and ZLNU were found to be main variables of importance, appearing in all three prediction models. Coarseness was correlated with SUV_max_ on PSF_wholeBody_ images (ρ = − 0.526, *p* = 0.005), whereas it was not on OSEM_breast_ (ρ = − 0.183, *p* = 0.244) and PSF_breast_ (ρ = − 0.244, *p* = 0.119) images. Moreover, the range of its values was higher on PSF_breast_ images as compared to OSEM_breast_, especially in small lesions (MTV < 3 ml).

**Conclusions:**

High-resolution breast PET acquisitions, applying both small-voxel matrix and PSF modelling, appeared to improve the characterisation of breast tumours.

**Electronic supplementary material:**

The online version of this article (10.1186/s13550-018-0466-5) contains supplementary material, which is available to authorized users.

## Background

Breast cancer is the most common type of cancer and the leading cause of death related to cancer in women worldwide [1]. It displays a large inter- and intra-tumour heterogeneity with a strong impact on patient management and outcome. Inter-patient tumoral heterogeneity can be reflected by actual staging systems and histopathological classifications that are predictors of patients’ outcomes and major determinants for treatment planning [2]. In the context of invasive breast cancer staging, 2-deoxy-2[^18^F]-fluoro-d-glucose (^18^F-FDG) positron emission tomography coupled with computed tomography (PET/CT) has shown its value for the detection of unexpected node involvements and/or distant metastasis [3]. Therefore, the European Society for Medical Oncology (ESMO) international consensus as well as the National Comprehensive Cancer Network (NCCN) guidelines recommend to consider the use of FDG PET-CT if available, instead of CT and bone scan for the initial staging of inoperable and non-metastatic locally advanced breast cancer (stage III with the exception of T3 N1) [4]. However, due to the high heterogeneity of breast cancers, FDG tumour uptake intensity measured as maximum standardised uptake value (SUV_max_) is highly variable, depending on multiple factors such as histological type, phenotypic type [5], proliferation index [6], histological grade and the presence of a P53 mutation [7] for example. However, SUV_max_ has been shown to be a prognostic index in invasive breast cancer [5]. More recently, PET textural features have emerged in the field of cancerology and have shown promising results in predicted response to treatment and/or patient survival in cervix, head and neck, lung and oesophageal cancer [8–16]. In breast cancers, heterogeneous tumour FDG uptake appeared to be frequent, especially in large tumours with intense FDG uptake [17]. Some studies have demonstrated that FDG breast tumour heterogeneity, based on single parameter or multi-feature signature, is significantly correlated with immunohistochemical factors and St Gallen’s subtypes [18–20]. Interestingly, these heterogeneity parameters were not correlated to SUV, meaning that they can surely provide additional information. However, these results are controversial because other studies did not find any ability of textural features (TFs) to discriminate between immunohistochemical subtypes [21, 22]. It is worth noticing that neither of these two studies used dedicated high-resolution images but images with standard 4 × 4 × 4 mm voxels. These findings thus suggest a potential role of textural features in breast cancer for non-invasive molecular subtype classification and subsequent patient prognosis stratification, but PET procedure seems to arise as a critical point in this field, especially when considering breast tumours that are usually small. Indeed, for such small lesions, it had already been demonstrated that small-voxel reconstruction and latest reconstruction algorithms bring better signal-to-noise ratio and could improve tumoral detection and the sensitivity of visual lymph node characterisation [23–25]. Therefore, the aim of this ancillary prospective clinical study is to compare different PET protocols with regard to their ability to discriminate between luminal versus non-luminal breast tumours.

## Material and methods

### Study population

This study is ancillary to a previous monocentric prospective study conducted by our team and approved by the local Ethics Committee (CPP Nord Ouest III, reference 2009-10) [23]. Informed and signed consent was obtained from all patients. Patients with newly diagnosed and histologically proven breast cancer for which breast surgery and axillary lymph node dissection was indicated were included from April 2009 to June 2012. All patients had a ^18^F-FDG PET/CT for initial staging of the disease.

### PET/CT acquisitions

PET imaging studies were performed on a Biograph TrueV (Siemens Medical Solutions). ^18^F-FDG injection was preceded by a 6-h fasting period and a 15-min rest in a warm room. Patients were scanned 60 min after ^18^F-FDG injection from the skull base to the mid-thighs (2 min 40 s per bed position for normal-weight patients (BMI ≤ 25 kg/m^2^) and 3 min and 40 s per bed position for patients with BMI > 25 kg/m^2^). Images were reconstructed using a point spread function (PSF) algorithm (HD; TrueX, Siemens Medical Solutions, 3 iterations and 21 subsets) with no post-filtering (PSF_WholeBody_) and a 168^2^ matrix size leading to a voxel size of 4.1 × 4.1 × 5.0 mm. A complementary high-resolution (HR) breast dedicated bed position (6 min per bed position) was performed just after the completion of the skull base to the mid-thighs acquisition. Images were reconstructed using the same protocol as above (PSF_breast_) and an ordered subset expectation maximization (OSEM) algorithm (four iterations, eight subsets) with a Gaussian post-filtering of 5 mm (OSEM_breast_) with a 512^2^ matrix size leading to voxels of 1.3 × 1.3 × 1.9 mm. Scatter and attenuation corrections were applied for both acquisitions.

### PET/CT images analysis

Injected dose, time between injection and acquisition and capillary glycaemia were recorded to seek for EANM recommendations fulfilment [26]. A single nuclear medicine physician drew volumes of interest (VOIs) encompassing the entire breast tumour on each PET acquisition using a PET edge method implemented in MIM software (MIM software, Cleveland, OH, US, version 5.6.5). In case of multiple lesions, only the biggest lesion was considered. To be close to real clinical practice, each PET dataset was contoured independently as it would have been done in a PET unit. The PET gradient method was used because it had been shown to be reproducible, little impacted by reconstruction type and have the ability to encompass the entire tumour by taking into account cold zones as opposed to threshold based VOIs [27]. Moreover, it is widely available. VOIs were subsequently saved as DICOM RT structures and then loaded in LifeX software [28] to extract SUV_max_, SUV_mean_, metabolic tumour volumes (MTV), total lesion glycolysis (TLG) and TFs parameters.

The following TFs were extracted:

- Homogeneity, energy, contrast, correlation, entropy, dissimilarity from grey level co-occurrence matrix (GLCM) that takes into account the arrangements of pairs of voxels
- Coarseness, contrast and busyness from neighbourhood grey-level different matrix (NGLDM) that corresponds to the difference of grey-level between one voxel and its 26 neighbours in 3 dimensions.
- SZE, LZE, LGZE, HGZE, SZLGE, SZHGE, LZLGE, LZHGE, GLNU, ZLNU, ZP (Table 1) from grey-level zone length matrix (GLZLM) that provides information on the size of homogeneous zones for each grey-level in three dimensions.

**Table 1:** Third-order textural features abbreviations

Variables	Definition
SZE	Short-zone emphasis
LZE	Long-zone emphasis
LGZE	Low grey-level zone emphasis
HGZE	High grey-level zone emphasis
SZLGE	Short-zone low grey-level zone emphasis
SZHGE	Short-zone high grey-level zone emphasis
LZLGE	Long-zone low grey-level zone emphasis
LZHGE	Long-zone high grey-level zone emphasis
GLNU	Grey-level non-uniformity
ZLNU	Zone length non-uniformity
ZP	Zone percentage

Absolute resampling using 64 grey levels between 0 and the maximum SUV units recorded for each reconstruction was used for all TFs: 27 for PSF_WholeBody_, 15 for OSEM_breast_ and 32 for PSF_breast_ leading to a size of bin of 0.4, 0.2 and 0.5, respectively [29, 30].

Coefficients of variation (CoV), measured in a 4 cm^3^ spherical VOI set in the descending thoracic aorta, were computed as follows for each reconstruction protocol:

$$ \mathrm{CoV}\kern0.5em =\kern0.75em \frac{\mathrm{standard}\ \mathrm{deviation}}{{\mathrm{SUV}}_{\mathrm{mean}}} $$

Further analyses were undergone. First, to assess the impact of quantification scaling, a supplemental analysis was undergone by using an upper SUV bound set to 32 for all 3 reconstructions leading to a size of bin of 0.5 for all reconstructions. Secondly, to assess the impact of the voxel size, a post-reconstruction resampling was applied to PSF_wholeBody_ and to PSF_breast_ images to obtain a 2 mm^3^ voxel size and a 4 mm^3^ voxel size, respectively.

### Statistical analysis

Quantitative data are presented as the median (interquartile range) or the mean (SD) when appropriate.

To compare PET metrics extracted from the three different reconstructions, non-parametric Friedman test with post-hoc test were used.

For each reconstruction protocol, a random forests (RF) method was used for building a prediction model for luminal versus non-luminal tumour classification. The method implemented classification and regression trees (CART, *n* = 100) and bootstrapping aggregating (bagging) method proposed by Breiman [31–33]. It allows studying the global heterogeneity of tumour rather than looking at individual features. For the validation, i.e. the training accuracy, the internal check in RF itself was used, based on the prediction error using the Out-Of-Bag (OOB) estimates of classification error: the smaller the OOB error rate, the better the reconstruction is able to classify between luminal and non-luminal tumours. Sensitivity (Se), specificity (Sp), positive predictive value (PPV), negative predictive value (NPV) and accuracy were computed. The importance of TFs in classification was assessed for each reconstruction protocol by measuring the mean decrease accuracy [34] of class prediction. Spearman coefficients were used to seek correlation between PET metrics of importance. Finally, the first three main PET metrics were considered for further paired comparison between reconstruction protocols using Friedman test with post-hoc test, Spearman correlation tests and ROC analyses.

Graph and statistical analysis were performed on XLSTAT Software (XLSTAT 2017: Data Analysis and Statistical Solution for Microsoft Excel. Addinsoft, Paris, France (2017)). For all statistical tests, a two-tailed *P* value of less than 0.05 was considered statistically significant.

## Results

### Patients and PET characteristics

Sixty-three patients were referred for the staging of breast carcinoma from April 2009 to June 2012. Sixteen patients were excluded from the analysis, for a final database of 47 patients (47 PET-CTs). The causes of exclusion were as follows: PET-CT not performed prior to surgery (*n* = 8), metastatic tumours on staging imaging (*n* = 4), missing data (*n* = 1) and breast lesions not visible on PET-CT (*n* = 3). The tumour types confirmed on histopathology included 38 infiltrating ductal carcinomas, 2 infiltrating lobular carcinomas, 5 mixed ductal/lobular infiltrating carcinomas, 1 tubular carcinoma and 1 infiltrating undifferentiated carcinoma. Patient characteristics are displayed in Table 2. Six patients (12.8%) had multiple lesions (range 2–4).

**Table 2:** Patients’ characteristics

Characteristics	Number of patients (*n* = 47)
Age (years), mean ± SD [min-max]	56.1 ± 12.3 [29–80]
Histologic type, *n* (%)	
Ductal	38 (80.8)
Lobular	2 (4.3)
Mixed type	5 (10.6)
Others	2 (4.3)
Estrogen receptor status, *n* (%)	
Positive	40 (85.1)
Negative	7 (14.9)
Progesterone receptor status, *n* (%)	
Positive	33 (70.2)
Negative	14 (29.8)
HER2 status	
Positive	6 (12.8)
Negative	41 (87.2)
Mitotic grade	
1–2	29 (61.7)
3	18 (38.3)
T, *n* (%)	
T1	13 (27.6)
T2	24 (51.1)
T3	10 (21.3)
*N*, *n* (%)	
0	14 (29.8)
1	24 (51.1)
2	5 (10.6)
3	4 (8.5)
AJCC stage, *n* (%)	
I	4 (8.5)
IIA	16 (34.0)
IIB	11 (23.4)
IIIA	12 (25.6)
IIIB	0 (0)
IIIC	4 (8.5)

*AJCC* American Joint Committee on Cancer

The mean injected dose was equal to 282.1 (67.5) MBq. The mean uptake time was 62.0 (7.7) min for the whole body and 81.9 (8.3) min for HR breast acquisitions.

### Lesions size: metabolic tumour volumes and number of voxels within the VOIs

**Fig. 1:**
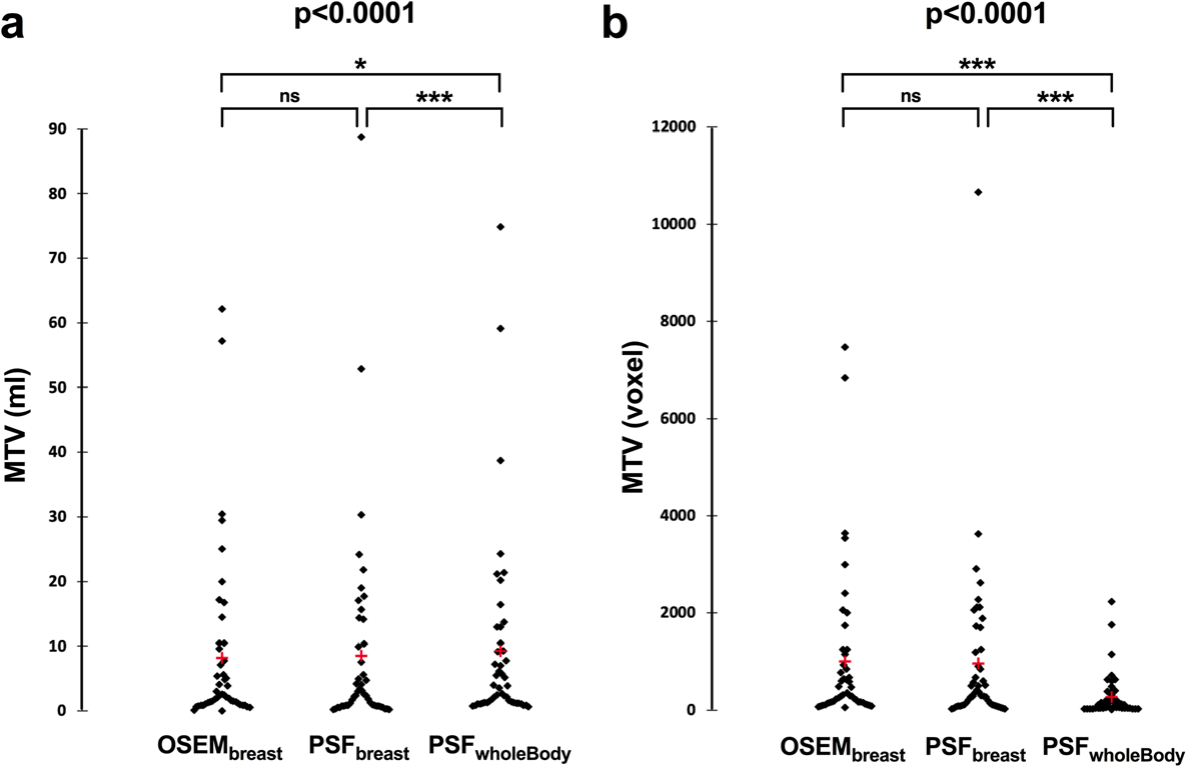
Paired comparison of OSEM_breast_, PSF_breast_ and PSF_wholeBody_ metabolic tumour volumes (**a**) and number of voxels within MTVs (**b**). Red cross represents the mean values. Legends for *p* values: ***< 0.001; **< 0.01; *< 0.05. *ns* non significant

Median metabolic tumour volumes (MTVs) (*n* = 47) were 3.62 (1.29–9.85), 2.43 (1.14–8.68), 2.43 (0.78–10.17) ml for PSF_WholeBody_, OSEM_breast_ and PSF_breast_, respectively (*p* < 0.00001). Thirty five patients (74.5%) had MTVs < 10 cm^3^ with at least one PET protocol. Dedicated HR breast acquisitions led to significantly smaller MTVs than PSF_wholeBody_ acquisitions for both OSEM_breast_ (*p* = 0.037) and PSF_breast_ (*p* < 0.0001). There was no significant difference between PSF_breast_ and OSEM_breast_ MTVs (*p* = 0.079) (Fig. 1a). The median numbers of voxels within VOIs were 130 (43–271), 316 (167–1042), 367 (107–1221) for PSF_WholeBody_, OSEM_breast_ and PSF_breast_, respectively (*p* < 0.0001). Dedicated HR breast acquisitions led to a significantly higher number of voxels than PSF_wholeBody_ acquisitions for both OSEM_breast_ (*p* < 0.0001) and PSF_breast_ (*p* < 0.0001) reconstructions. There was no significant difference between PSF_breast_ and OSEM_breast_ numbers of voxels (*p* = 0.062) (Fig. 1b). To be analysed in LifeX software, MTVs should contain at least 64 voxels. Therefore, 28 (59%), 46 (98%) and 42 (89%) patients were exploitable when using PSF_WholeBody_, OSEM_breast_ and PSF_breast_ reconstructions, respectively. Of note, due to a very low MTV, only one patient was not analysable by all three reconstructions and therefore was not included in the subsequent statistical analysis. She was a 72-year-old woman presenting a luminal A (ER+; PR+, HER2−, grade 1) breast tumour classified T1N1M0.

### Prediction accuracies for luminal status tumours classification and variables of importance identification for PSF_wholeBody_, PSF_breast_ and OSEM_breast_ PET protocols

**Fig. 2:**
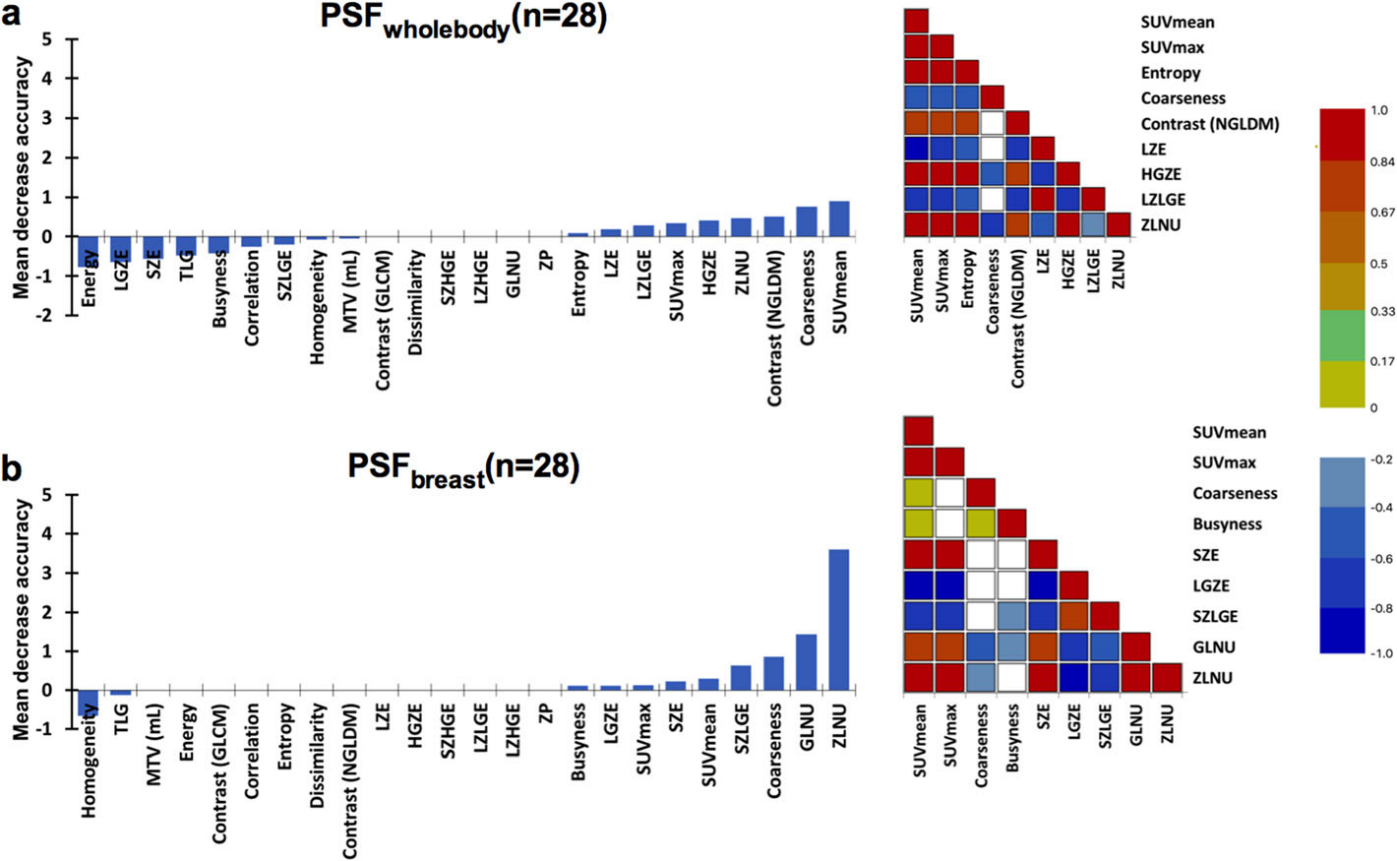
Left panels display the mean decrease accuracy of textural features values and right panels display Spearman correlation matrixes of all PET metrics found to have positive mean decrease accuracy, whatever the value for PSF_wholeBody_ (**a**) and PSF_breast_ (**b**) reconstructions. For Spearman correlation matrixes, the blue colour corresponds to a correlation close to − 1 and the red colour corresponds to a correlation close to 1. The green corresponds to a correlation close to 0

When matched comparing the 28 patients analysed with PSF_wholeBody_ and PSF_breast_ (22 luminal and 6 non-luminal tumours), PSF_wholeBody_ showed higher OOB estimates of classification error than PSF_breast_ with values equal to 32.1% and 25.0%, respectively. Accuracy, Se, Sp, PPV and NPV are displayed in Table 3 and variables of importance for both PET protocols are displayed on Fig. 2. Interestingly, both protocols found coarseness and ZLNU to be variables of importance. However, coarseness was negatively correlated with SUV_max_ and SUV_mean_ on PSF_wholeBody_ images (ρ = − 0.526, *p* = 0.005 and ρ = − 0.406, *p* < 0.033, respectively), whereas it was not on PSF_breast_ images (ρ = − 0.093, *p* = 0.636 and ρ = 0.139, *p* < 0.479, respectively). ZLNU was correlated with SUV_max_ and SUV_mean_ on both PSF_wholeBody_ and PSF_breast_ images (ρ = 0.928, *p* < 0.0001 and ρ = 0.962, *p* < 0.0001, respectively for SUV_max_ correlation). Noticeably, variables of importance mean decrease accuracies were globally lower for PSF_wholeBody_ images as compared to PSF_breast_ images. Moreover, all textural features were highly correlated with SUV_max_ and SUV_mean_ values for PSF_wholeBody_ images, whereas correlations were fewer and lower for PSF_breast_ images (Fig. 2). Concerning images noise, there was no significant difference between PSF_wholeBody_ and PSF_breast_ images with a mean CoV of 0.175 (0.030) and 0.189 (0.031), respectively (*p* = 0.087). Moreover, coarseness was not correlated to noise: ρ = − 0.029, *p* = 0.883 for PSF_wholeBody_ and ρ = 0.190, *p* = 0.330 for PSF_breast_.

**Table 3:** Reconstruction protocols classification performances regarding tumour luminal status detection

		Accuracy (%)	Se (%)	Sp (%)	PPV (%)	NPV (%)
Comparison of 28 patients	PSF_wholeBody_	67.8	86.4	0.0	76.0	0.0
	PSF_breast_	75.0	90.9	16.7	80.0	33.3
Comparison of 42 patients	OSEM_breast_	73.8	85.7	14.3	83.3	16.7
	PSF_breast_	85.7	94.3	42.9	89.2	60.0

*Se* sensitivity, *Sp* specificity, *PPV* positive predictive value, *NPV* negative predictive value

When applying a size of bin equal to 0.5 on PSF_wholeBody_ images to meet the quantification scale of PSF_breast_, the OOB estimates of classification error went from 32.1 to 28.6%, still higher than PSF_breast_ OOB estimates of classification error. Variables of importance and their correlations are displayed on Additional file 1: Figure S1a.

After applying to PSF_wholeBody_ images a 2 mm^3^ post-reconstruction voxel resampling, the OOB estimates of classification error remained stable, equal to 32.1%. Variables of importance and their correlations for both protocols are displayed on Additional file 2: Figure S2a. Interestingly, there was no more correlation between SUV_max_ and coarseness values on PSF_wholeBody_ images after post-reconstruction voxel resampling: ρ = − 0.276, *p* = 0.155.

**Fig. 3:**
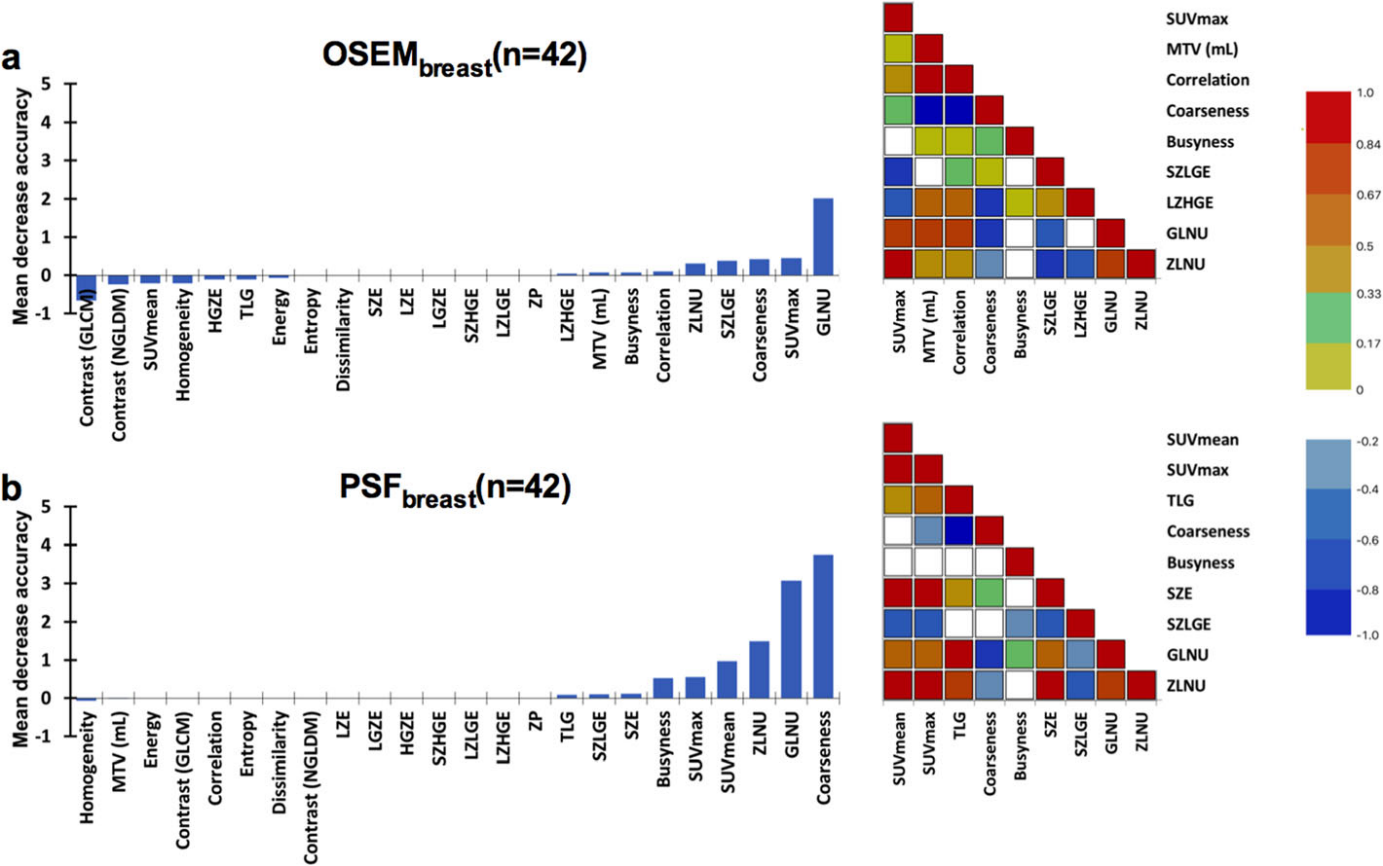
Left panels display the mean decrease accuracy of textural features values and right panels display Spearman correlation matrixes of all PET metrics found to have positive mean decrease accuracy, whatever the value for OSEM_breast_ (**a**) and PSF_breast_ (**b**) reconstructions. For Spearman correlation matrixes, the blue colour corresponds to a correlation close to − 1 and the red colour corresponds to a correlation close to 1. The green corresponds to a correlation close to 0

When matched comparing the 42 patients analysed with both OSEM_breast_ and PSF_breast_ (35 luminal and 7 non-luminal tumours), PSF_breast_ showed higher classification accuracy and lower OOB estimates of classification error than OSEM_breast_. OOB estimates were equal to 26.2% and 14.3% when using OSEM_breast_ and PSF_breast_, respectively. Accuracy, Se, Sp, PPV and NPV are displayed in Table 3. Both protocols showed high sensitivity but low specificity for the luminal status detection: the best specificity was obtained using PSF_breast_ with a value equal to 42.9%. Figure 3 displays variables of importance for both PET protocols and demonstrates that coarseness and ZLNU were again variables of importance with both protocols as well as GLNU, SZLGE and busyness. GLNU and ZLNU were found to be significantly positively correlated with each other (*p* < 0.0001) and with SUV_max_ (p < 0.0001) on both protocols. Coarseness was not correlated with SUV_max_ with ρ equal to − 0.183 (*p* = 0.244) for OSEM_breast_ and − 0.244 (*p* = 0.119) for PSF_breast_. Concerning images noise, there was no significant difference between OSEM_breast_ and PSF_breast_ images with a mean CoV of 0.175 (0.030) and 0.189 (0.031), respectively (*p* = 0.087). Moreover, coarseness was not correlated to noise: ρ = − 0.029, *p* = 0.883 for PSF_wholeBody_ and ρ = 0.190, *p* = 0.330 for PSF_breast_.

When applying a size of bin equal to 0.5 on OSEM_breast_ images to meet the quantification scale of PSF_breast_, the OOB estimates of classification error decreased, equal to 21.4%, but were still higher than PSF_breast_ OOB estimates of classification error. Variables of importance and their correlations are displayed on Additional file 1: Figure S1b.

After applying to PSF_breast_ images a 4 mm^3^ post-reconstruction voxel resampling, the OOB estimates of classification error increased moderately, equal to 26.2%. Variables of importance and their correlations for both protocols are displayed on Additional file 2: Figure S2b. Of note, when applying a 4 mm^3^ post-reconstruction voxel resampling on PSF_breast_ images, coarseness was then correlated to SUV_max_ values: ρ = − 0.321, *p* = 0.05.

### Comparison of coarseness, GLNU and ZLNU values obtained from PSF_breast_ and OSEM_breast_ protocols using adapted SUV_max_ bounds for each reconstruction to quantify textural features

**Fig. 4:**
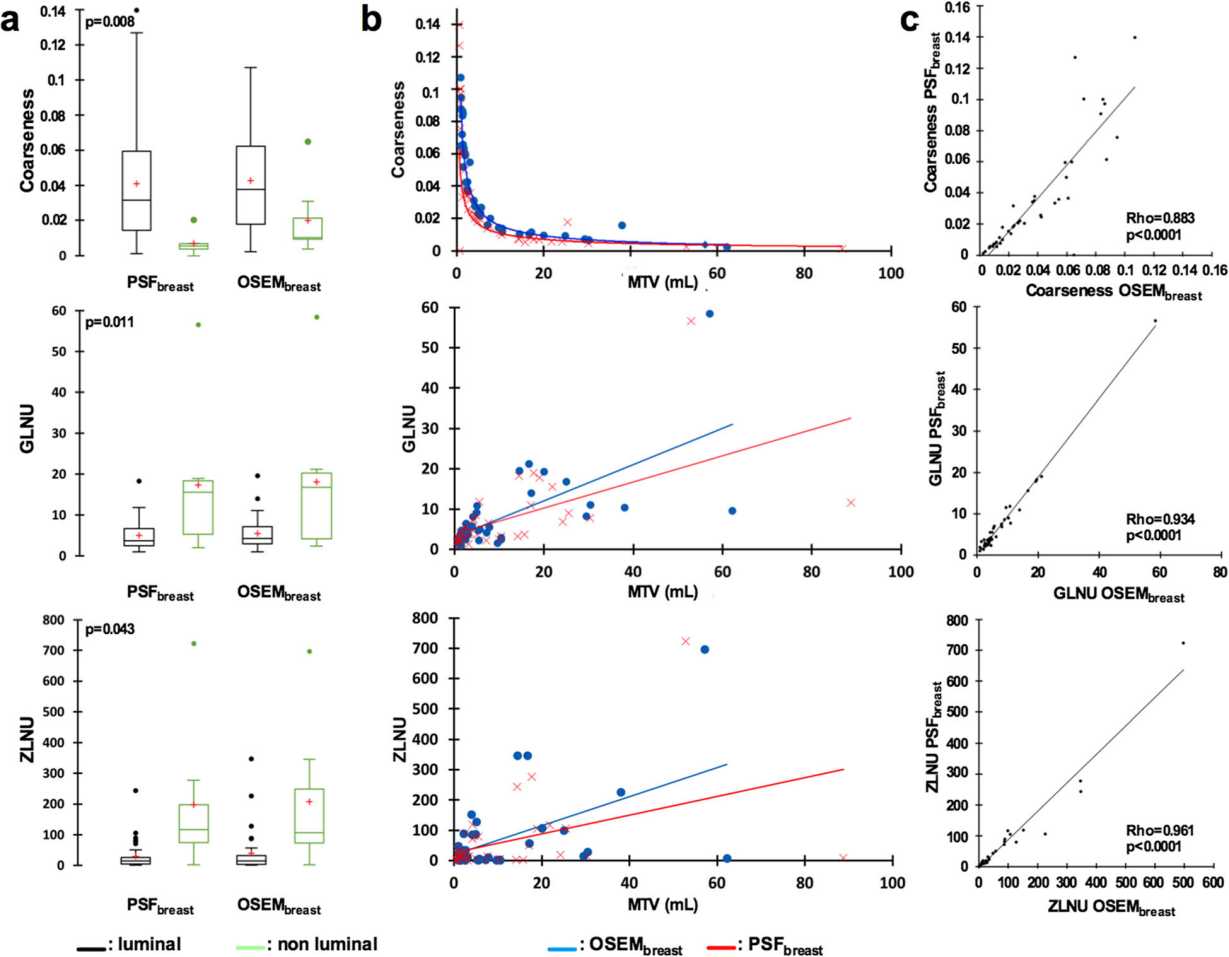
Comparison of coarseness, GLNU and ZLNU values extracted from PSF_breast_ and OSEM_breast_ images: box plots (**a**) correlation with MTV (ml) (**b**) and correlation between reconstruction protocols (**c**). Red cross in box plots represents the mean values and circle extreme values

**Fig. 5:**
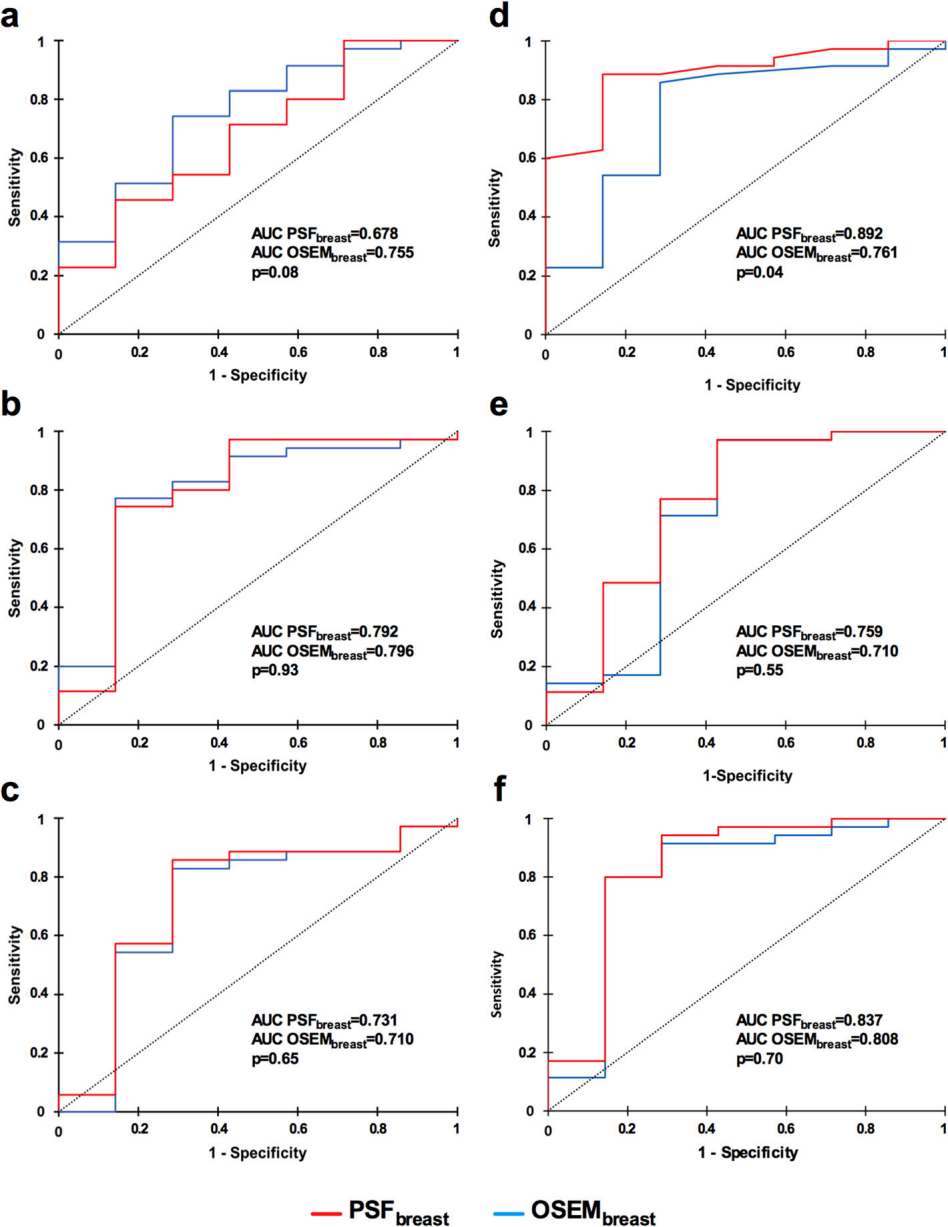
ROC curves for SUV_mean_ (**a**), SUV_max_ (**b**), MTV (**c**), coarseness (**d**), GLNU (**e**) and ZLNU (**f**) values. The blue line corresponds to OSEM_breast_ and the red one to PSF_breast_ reconstruction

**Fig. 6:**
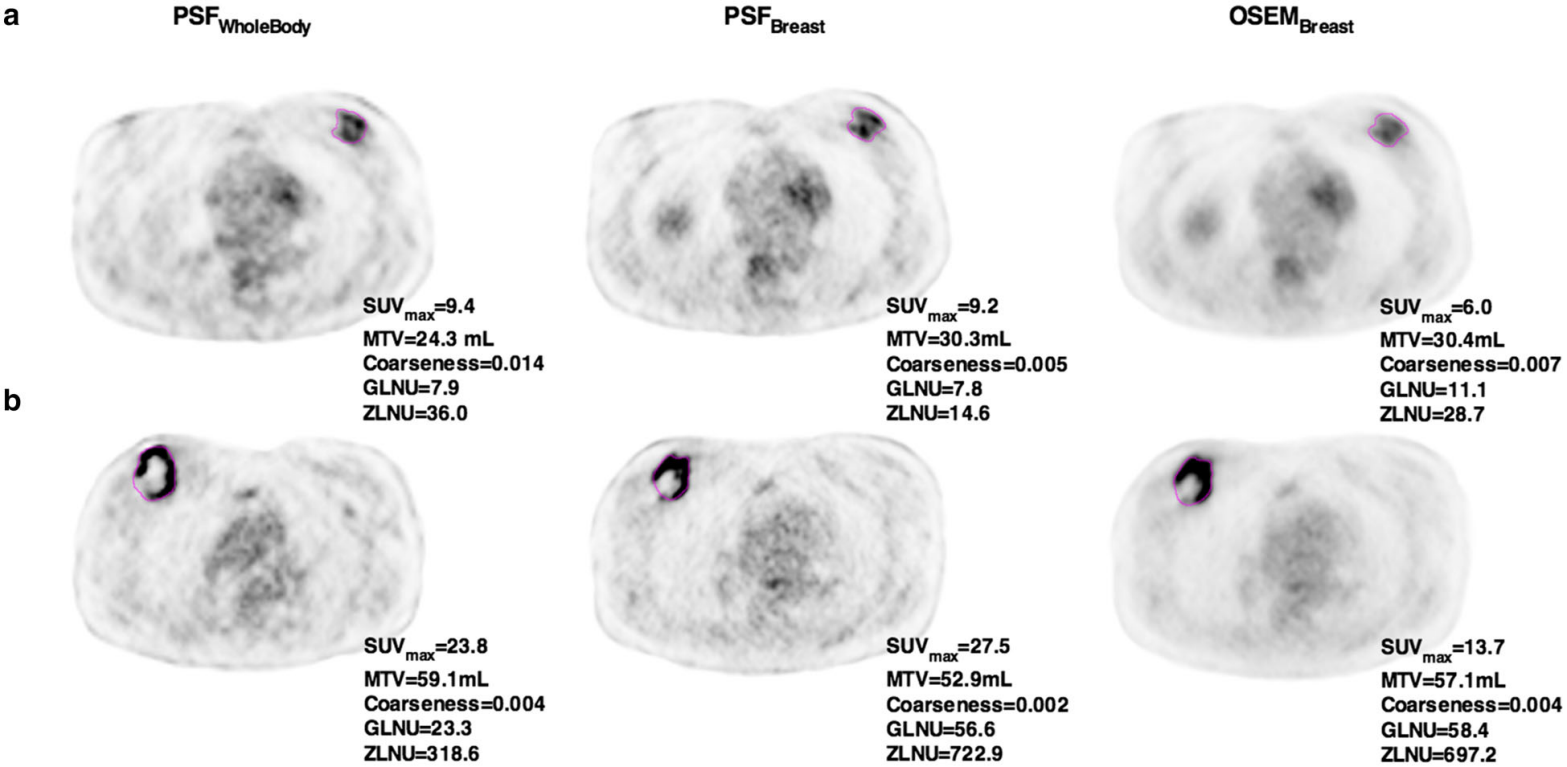
Representative images of one luminal (**a**) and one non-luminal (**b**) breast tumour. The luminal tumour was a luminal B HER2 negative T3N1M0 ductal carcinoma. The non-luminal tumour was a T2N1M0 triple-negative ductal carcinoma. Images were scaled on the same maximum value (SUV = 5)

Paired comparison of PSF_breast_ and OSEM_breast_ reconstructions found significant differences between coarseness, GLNU and ZLNU values (Fig. 4a). Interestingly, the range of coarseness values was wider when using PSF_breast_ especially for the smallest lesions, whereas it was quite similar between PSF_breast_ and OSEM_breast_ for GLNU and ZLNU values (Fig. 4b). However, PSF_breast_ and OSEM_breast_ coarseness, GLNU and ZLNU values were highly correlated (Fig. 4c). Coarseness displayed the lowest ρ value: 0.883 (*p* < 0.0001) with a dispersion of coarseness values between PET protocols occurring for coarseness values superior to 0.04 corresponding to the smallest lesions (MTV < 3 ml). On the contrary, GLNU and ZLNU seem to have the same distribution whatever the protocols and the MTV considered. Moreover, there was no difference between PSF_breast_ and OSEM_breast_ areas under the ROC for the luminal versus non-luminal status determination with GLNU values and ZLNU values, whereas the area under the ROC with PSF_breast_ coarseness values was significantly higher than that of OSEM_breast_ coarseness values (Fig. 5). Representative images of one luminal and one non-luminal breast tumours are shown on Fig. 6.

## Discussion

As expected, there was a limited number of analysable tumours when using PSF_wholeBody_. Although this reconstruction led to larger MTVs, the number of voxels within MTVs was very low as compared to OSEM_breast_ and PSF_breast_ and thus led to 19 patients (40.4%) being non-exploitable. On the contrary, OSEM_breast_ and PSF_breast_ led to smaller MTVs but a higher number of voxels and therefore allowed studying nearly all patients: 98% for OSEM_breast_ and 89% for PSF_breast_.

On matched comparison, PSF_breast_ reconstruction presented better abilities than PSF_wholeBody_ and OSEM_breast_ for the classification of luminal versus non-luminal breast tumours with an accuracy reaching 85.7%. Using the same heterogeneity quantification scale for all three reconstructions, PSF_breast_ still showed higher abilities than others reconstructions. Noticeably, it displayed a high sensitivity but low specificity for the detection of luminal status. Coarseness and ZLNU were the only PET TFs identified as important classification variables by all three reconstruction models. But on PSF_wholeBody_ images, coarseness was highly correlated with SUV_max_, whereas it was not for HR breast protocols. Moreover, correlation between SUV_max_ and coarseness values seems to be linked to voxel size as it disappeared after applying a 2 mm^3^ post-reconstruction voxel resampling to PSF_wholeBody_ images and appeared after applying a 4 mm^3^ post-reconstruction voxel resampling to PSF_breast_ images. The numerous and strong correlations of TFs with SUV_max_ observed on PSF_wholeBody_ suggested that PET metrics extracted from PSF_wholeBody_ may have less additional information over conventional PET indices. One could consider that the delay between whole body acquisition and the dedicated breast acquisition may have influenced our results. However, this delay was around 20 min and we feel unlikely that it influenced TFs values, as opposed to a previous study in which a second examination was performed 3 h after injection, with a mean time of 127 min between the two phases [35]. Considering image noise, one could have expected that using a small-voxel matrix would have led to higher noise in PSF_breast_ images as compared to PSF_wholeBody_. However, no significant difference in CoV was observed in the present study among all reconstruction protocols, but the small matrix size may have been counterbalanced by a longer acquisition time.

Among HR breast bed position, PSF reconstruction appeared to be more discriminative for luminal versus non-luminal status than OSEM reconstruction. This is in accordance with our previous publication [36] that compared those two types of reconstruction. Regarding PET metrics extracted from NGLDM matrix and especially coarseness, there was higher values dispersion with PSF_breast_ reconstruction, especially for small lesions and a better area under the ROC for luminal versus non-luminal status determination. Besides, this metric was not correlated to SUV_max_ suggesting that it could provide additional information. Considering TFs extracted from GLZLM, especially ZLNU and GLNU, no difference was found in the dispersion of these TFs values between PSF_breast_ and OSEM_breast_ reconstructions. As coarseness, GLNU and ZLNU were not explored in previous studies, no comparison can be made [17–22].

Concerning heterogeneity quantification process, the main analysis was designed in order to obtain data as close as possible to what could have been done in routine clinical practice, for example in PET units using different reconstruction algorithms. To this end, VOIs and SUV bounds were adapted to each reconstruction independently. To test the influence of quantification scale, a supplemental analysis was made using same SUV bounds for all reconstructions leading to same bin widths and showed no major change as compared to the first analysis. However, as SUV are highly reconstruction-dependent, with for example a mean percentage difference that could reach 66% between OSEM and PSF reconstructions [23], we firmly believe that SUV bounds have to be adapted specifically to the reconstruction of interest. When it comes to VOIs delineation, an appropriate VOI for each reconstruction seems more relevant to answer the question of the influence of reconstruction on FDG radiomics. Indeed, using the same volume of interest for all reconstructions is never meant to happen in clinical practice. Besides, using same VOIs or independent VOIs between different reconstructions showed almost no influence on a panel of second- and third-order textural features in a previous study [36]. Finally, small-voxel post-reconstruction resampling did not provide better capabilities in terms of histological classification and therefore seems to offer no additional information.

This study had some limitations. First of all, although random forests allowed matched comparison of datasets, it surely did not give definitive results concerning the ability of TFs in discriminating histological characteristics of breast tumours in view of the limited number of patients. The limited number of patients did not allow us to consider all histological tumour subtypes and therefore the discriminative power of TFs was restricted to luminal versus non-luminal tumours. However, the aim of the present study was not to have definitive results concerning PET abilities for histological discrimination. It demonstrated that a combination of PSF modelling and small-voxel reconstruction seems to be the best strategy to obtain additional information over conventional PET metrics and should be used when characterising the intra-tumoral FDG heterogeneity of breast cancers. These results are in line with previous publications using a breast-dedicated PET system, small-voxels and/or new generation reconstruction algorithms with time-of-flight, which found that FDG breast tumour heterogeneity was significantly correlated with immunohistochemical factors and St Gallen’s subtypes [18–20], whereas those using OSEM reconstruction with 4 × 4 × 4 mm voxels did not find any association [21, 22].

## Conclusions

High-resolution breast PET acquisitions, applying both small-voxel matrix and PSF modelling, appeared to be necessary to improve the characterisation of breast tumours, especially when seeking a link between ^18^F-fluorodeoxyglucose heterogeneity and histological characteristics in breast cancer.

## Additional files

Additional file 1: **Figure S1.** Impact of quantification scale. Left panels display the mean decrease accuracy of textural features values and right panels display Spearman correlation matrixes of all PET metrics found to have positive mean decrease accuracy, whatever the value for PSF_wholeBody_ (a) and OSEM_breast_ (b) reconstructions. SUV bounds were set to 0–32 leading to a size of bin of 0.5 for both reconstructions. For Spearman correlation matrixes the blue colour corresponds to a correlation close to − 1 and the red colour corresponds to a correlation close to 1. The green corresponds to a correlation close to 0. (TIFF 5689 kb)

Additional file 2: **Figure S2.** Impact of voxels post-reconstruction resampling. Left panels display the mean decrease accuracy of textural features values and right panels display Spearman correlation matrixes of all PET metrics found to have positive mean decrease accuracy as well as SUV_max_ and coarseness for PSF_wholeBody_ after a 2mm^3^ voxels resampling (a) and PSF_breast_ after a 4mm^3^ voxels resampling (b) reconstructions. For Spearman correlation matrixes the blue colour corresponds to a correlation close to − 1 and the red colour corresponds to a correlation close to 1. The green corresponds to a correlation close to 0. (TIFF 6529 kb)

## Abbreviations

BMI: Body mass index
CART: Classification and regression trees
CoV: Coefficient of variation
CPP: Comité de Protection des Personnes
DICOM: Digital Imaging and Communications in Medicine
ER: Estrogen receptors
ESMO: European Society for Medical Oncology
FDG: Fluorodeoxyglucose
GLCM: Grey level co-occurrence matrix
GLNU: Grey-level non-uniformity for zone
GLZLM: Grey-level zone length matrix
HER2: Human epidermal growth factor receptor-2
HGZE: Low grey-level zone emphasis
LGZE: Low grey-level zone emphasis
LZE: Long-zone emphasis
LZHGE: Long-zone high grey-level emphasis
LZLGE: Long-zone low grey-level emphasis
MTV: Metabolic tumour volume
NCCN: National comprehensive cancer network
NGLDM: Neighbourhood grey-level different matrix
NPV: Negative predictive value
OOB: Out-of-bag
OSEM: Ordered subset expectation maximization
PET: Positron emission tomography
PPV: Positive predictive value
PR: Progesterone receptors
PSF: Point spread function
Se: Sensitivity
Sp: Specificity
SZE: Short-zone emphasis
SZHGE: Short-zone high grey-level emphasis
SZLGE: Short-zone low grey-level emphasis
TF: Textural feature
VOI: Volume of interest
ZLNU: Zone length non-uniformity
ZP: Zone percentage
