# Supplementary figures and images for: Implications of reconstruction protocol for histo-biological characterisation of breast cancers using FDG-PET radiomics
Source: EJNMMI Res. 2018 Dec 29;8:114. doi: 10.1186/s13550-018-0466-5 (PMC6311169; doi:10.1186/s13550-018-0466-5)

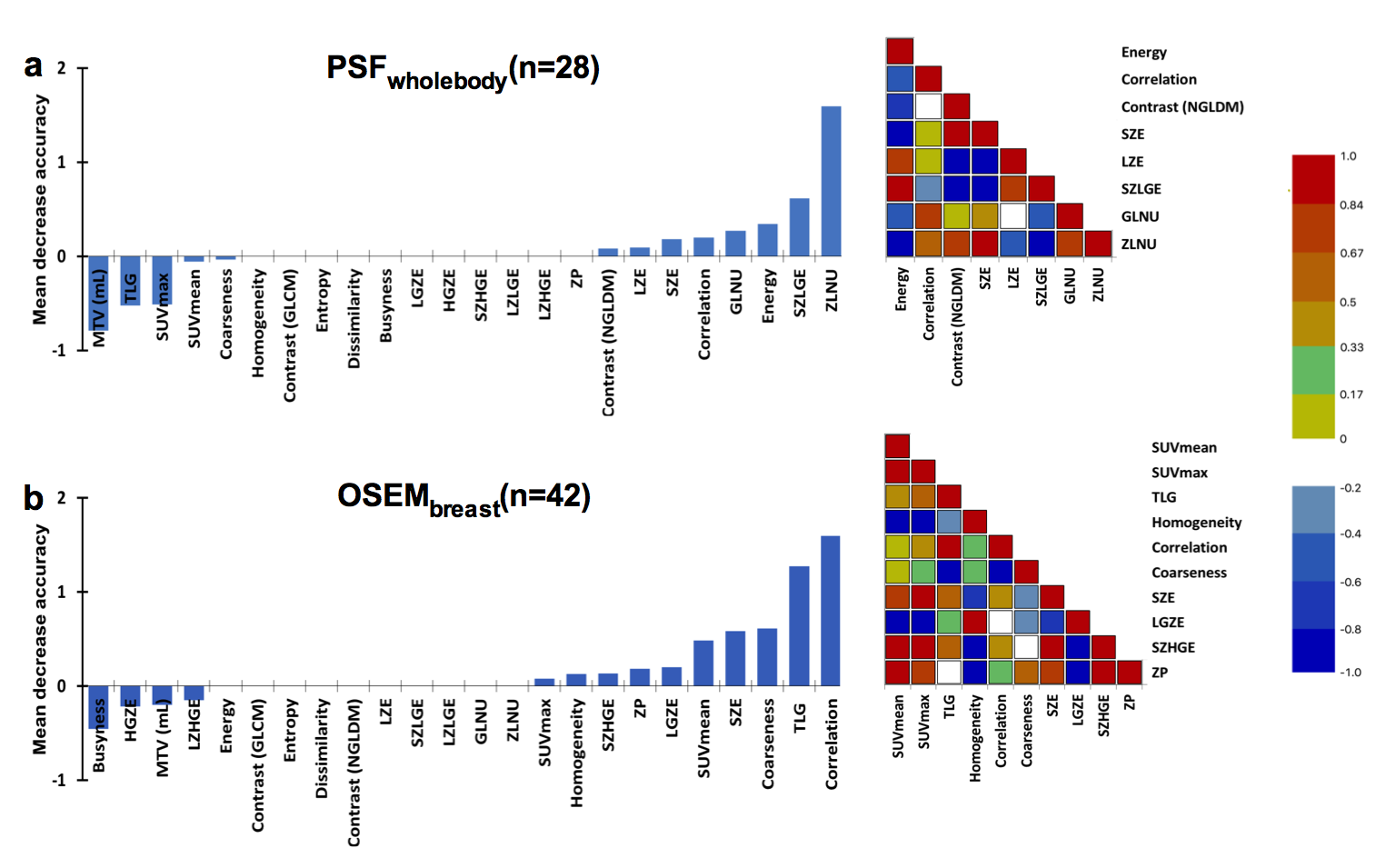

Supplement: Supplementary file 1 — Figure S1. Impact of quantification scale. Left panels display the mean decrease accuracy of textural features values and right panels display Spearman correlation matrixes of all PET metrics found to have positive mean decrease accuracy, whatever the value for PSFwholeBody (a) and OSEMbreast (b) reconstructions. SUV bounds were set to 0–32 leading to a size of bin of 0.5 for both reconstructions. For Spearman correlation matrixes the blue colour corresponds to a correlation close to − 1 and the red colour corresponds to a correlation close to 1. The green corresponds to a correlation close to 0. (TIFF 5689 kb) [file 13550_2018_466_MOESM1_ESM.tiff]

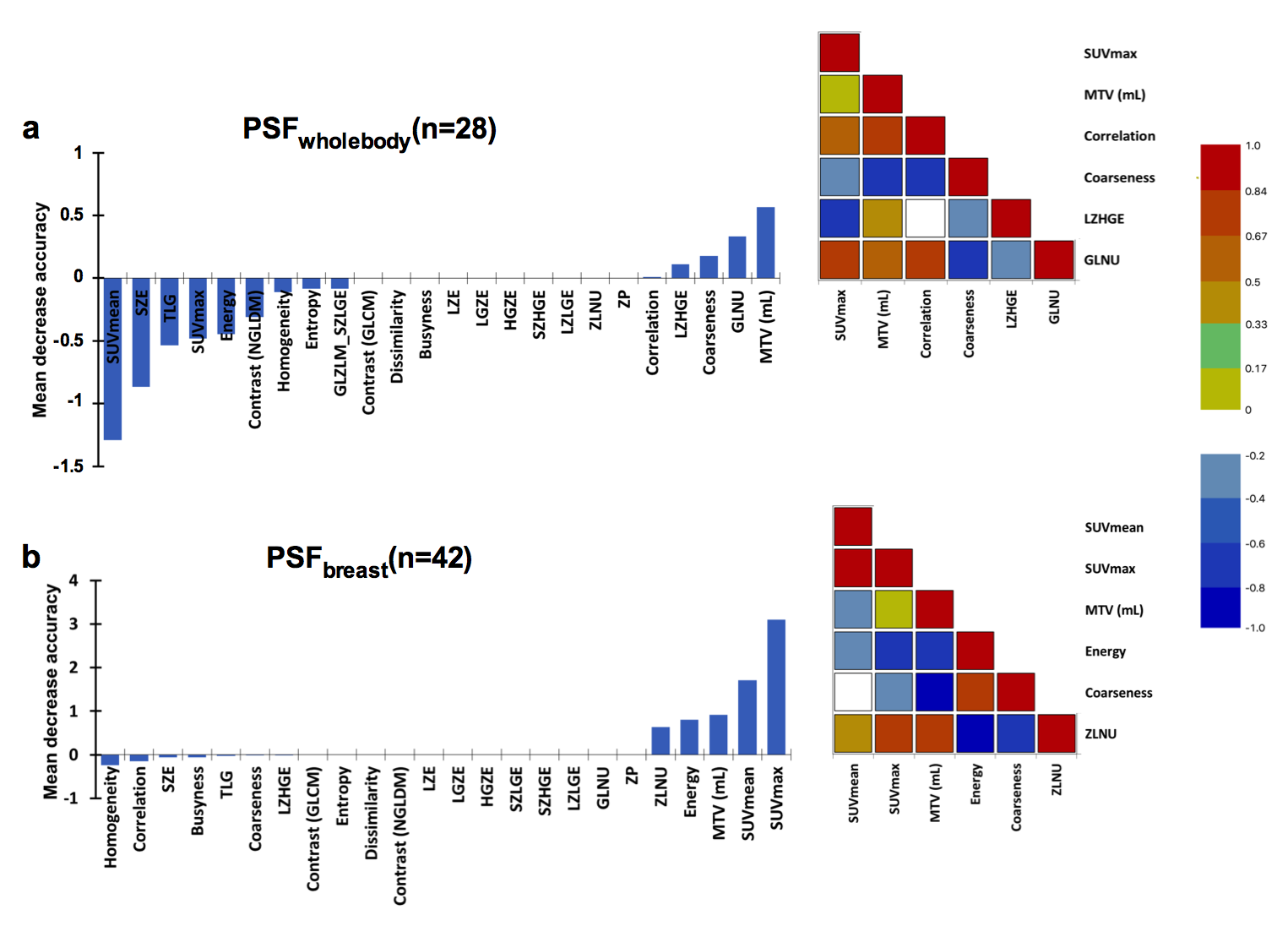

Supplement: Supplementary file 2 — Figure S2. Impact of voxels post-reconstruction resampling. Left panels display the mean decrease accuracy of textural features values and right panels display Spearman correlation matrixes of all PET metrics found to have positive mean decrease accuracy as well as SUVmax and coarseness for PSFwholeBody after a 2mm3 voxels resampling (a) and PSFbreast after a 4mm3 voxels resampling (b) reconstructions. For Spearman correlation matrixes the blue colour corresponds to a correlation close to − 1 and the red colour corresponds to a correlation close to 1. The green corresponds to a correlation close to 0. (TIFF 6529 kb) [file 13550_2018_466_MOESM2_ESM.tiff]
